# Seed/catalyst-free vertical growth of high-density electrodeposited zinc oxide nanostructures on a single-layer graphene

**DOI:** 10.1186/1556-276X-9-95

**Published:** 2014-02-26

**Authors:** Nur Suhaili Abd Aziz, Mohamad Rusop Mahmood, Kanji Yasui, Abdul Manaf Hashim

**Affiliations:** 1 Malaysia-Japan International Institute of Technology, Universiti Teknologi Malaysia, Jalan Semarak, Kuala Lumpur 54100, Malaysia; 2 Faculty of Electrical Engineering, Universiti Teknologi MARA, Shah Alam, Selangor 40540, Malaysia; 3 Department of Electrical Engineering, Nagaoka University of Technology, Kamitomioka-machi, Nagaoka, Niigata 940-2137, Japan; 4 MIMOS Berhad, Technology Park Malaysia, Kuala Lumpur 57000, Malaysia

**Keywords:** Electrochemical deposition, Graphene, Zinc oxide, One-dimensional nanostructure

## Abstract

We report the seed/catalyst-free vertical growth of high-density electrodeposited ZnO nanostructures on a single-layer graphene. The absence of hexamethylenetetramine (HMTA) and heat has resulted in the formation of nanoflake-like ZnO structure. The results show that HMTA and heat are needed to promote the formation of hexagonal ZnO nanostructures. The applied current density plays important role in inducing the growth of ZnO on graphene as well as in controlling the shape, size, and density of ZnO nanostructures. High density of vertically aligned ZnO nanorods comparable to other methods was obtained. The quality of the ZnO nanostructures also depended strongly on the applied current density. The growth mechanism was proposed. According to the growth timing chart, the growth seems to involve two stages which are the formation of ZnO nucleation and the enhancement of the vertical growth of nanorods. ZnO/graphene hybrid structure provides several potential applications in electronics and optoelectronics such as photovoltaic devices, sensing devices, optical devices, and photodetectors.

## Background

In recent years, graphene/semiconductor nanocrystal hybrid structure is particularly interesting because nanostructures, such as nanowires, nanorods, nanoneedles, nanosheets and nanowalls, can offer additional functionality to graphene for realizing advanced nanoscale electronics and optoelectronic applications in photovoltaics, nanogenerators, field emission devices, sensitive biological and chemical sensors, and efficient energy conversion and storage devices [1-5]. This is due to their high aspect ratio, high thermal and mechanical stability, extremely large surface-to-volume ratio, and high porosity [6-9]. Graphene has a great potential for novel electronic devices because of their extraordinary electrical, thermal, and mechanical properties, including a carrier mobility exceeding 10^4^ cm^2^/Vs and a thermal conductivity of 10^3^ W/mK [10-13]. Therefore, with the excellent electrical and thermal characteristics of graphene layers, growing semiconductor nanostructures and thin films on graphene layers would enable their novel physical properties to be exploited in diverse sophisticated device applications. Recently, several graphene/semiconductor nanocrystals have been successfully synthesized that show desirable combinations of these properties not found in the individual components. One-dimensional zinc oxide (ZnO) semiconducting nanostructures are considered to be important multifunctional building blocks for fabricating various nanodevices [14,15]. Since graphene is an excellent conductor and a transparent material, the hybrid structure of ZnO/graphene shall lead to several device applications not only on silicon (Si) substrate but also on other insulating substrates such as glass and flexible plastic. Owing to the unique electronic and optical properties of ZnO nanostructures, such hybrid structure can be used for sensing devices [16,17], ultraviolet (UV) photodetectors [18], solar cells [19], and light-emitting diodes (LED) [20].

There are several potential methods to grow ZnO on graphene which can be categorized into vapor-phase and liquid-phase methods. The vapor phase method is likely to involve high-temperature process and is also considered as a high-cost method [2,21]. Also, since the process requires oxygen (O_2_), the possibility of graphene to be oxidized or etched out during the growth is high since the oxidation of graphene is likely to occur at temperature as low as 450°C [22]. The liquid-phase method seems to be a promising method to grow graphene at low temperature with good controllability in terms of growth rates and structure dimensions.

Up to date, only two methods have been reported on the growth of seed/catalyst-free ZnO nanostructure on graphene via low-temperature liquid-phase method. Kim et al*.* reported the growth of ZnO nanorods on graphene without any seed layer by hydrothermal method, but the obtained results show low density of nanostructures [23]. Xu et al*.* reported the seedless growth of ZnO nanotubes and nanorods on graphene by electrochemical deposition [24,25]. They reported the growth of highly dense ZnO nanostructures by using solely zinc nitrate as the electrolyte with the introduction of oxidation process of graphene prior to the actual growth. In this paper, we report the seed/catalyst-free vertical growth of ZnO nanostructures on graphene by a single-step cathodic electrochemical deposition method. The term ‘seed/catalyst-free’ refers to the omission of predeposition of ZnO seed layer and any kind of catalyst by other processes. A highly dense vertically aligned ZnO nanostructure on a single-layer (SL) graphene was successfully grown.

## Methods

**Figure 1:**
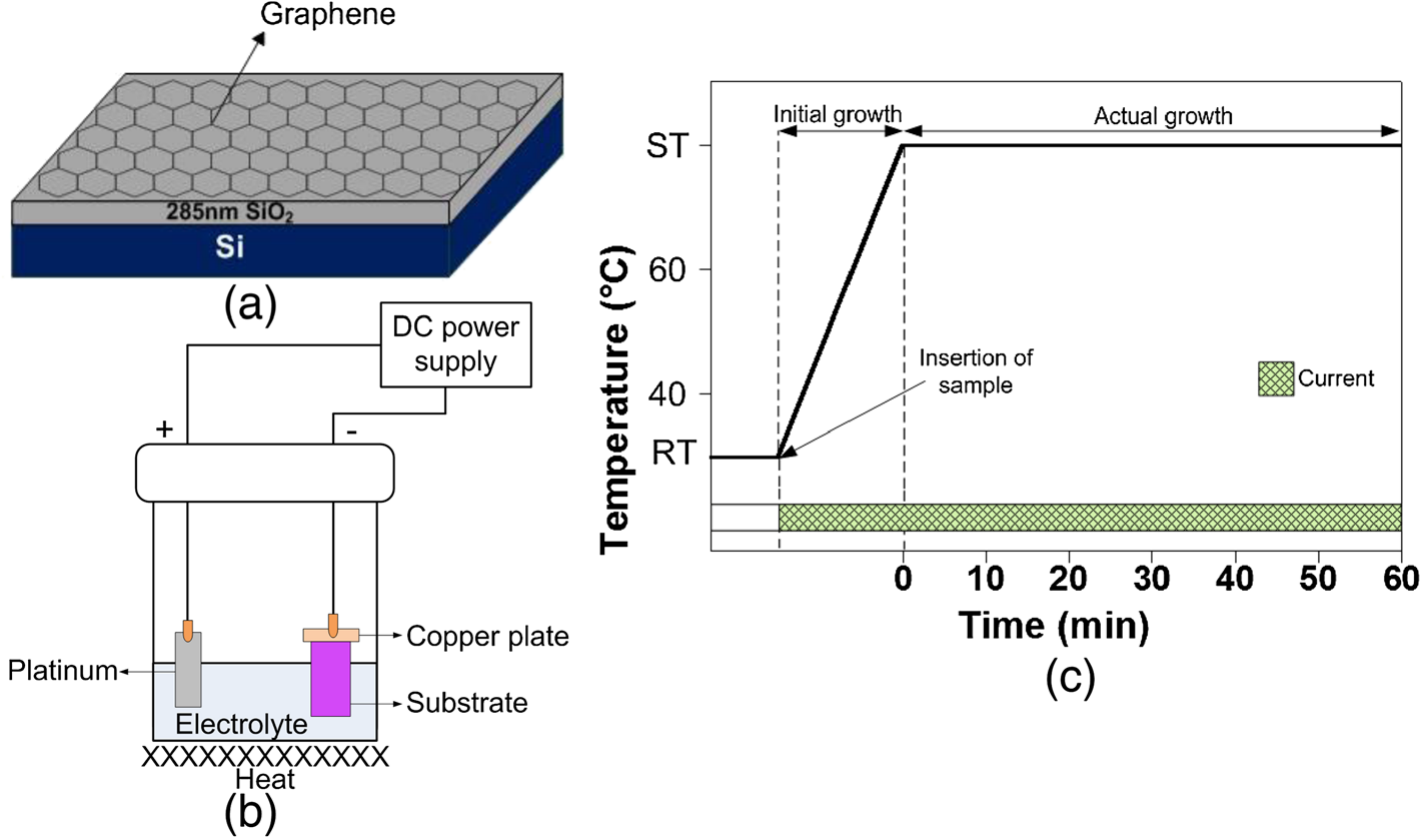
**Schematics and time chart. (a)** Schematic of substrate with single-layer graphene, **(b)** schematic of electrochemical setup, and **(c)** time chart for electrochemical process.

Figure 1a shows the schematic of chemical vapor deposition (CVD)-grown SL graphene on silicon dioxide (SiO_2_)/Si substrate (Graphene Laboratories Inc., Calverton, NY, USA). The growth of the ZnO nanostructures on graphene/SiO_2_/Si was carried out by a cathodic electrochemical deposition in 50 mM of zinc nitrate hexahydrate (Zn(NO_3_)_2_ · 6H_2_O, ≥99.0% purity; Sigma-Aldrich, St. Louis, MO, USA) and hexamethylenetetramine (HMTA, C_6_H_12_N_4_, ≥99.0% purity, Sigma-Aldrich). As shown in Figure 1b, platinum (Pt) wire acted as an anode (counter electrode), while the graphene acted as a cathode. Both anode and cathode were connected to the external direct current (DC) power supply. Different current densities of -0.1, -0.5, -1.0, -1.5, and -2.0 mA/cm^2^ were applied. The sample was inserted into the electrolyte from the beginning of the process before this electrolyte was heated up from room temperature (RT) to 80°C. The growth was done for 1 h, counted when the electrolyte temperature reached 80°C or the set temperature (ST). Such temperature was chosen since the effective reaction of zinc nitrate and HMTA takes place at temperature above 80°C. After 1 h, the sample was removed immediately from the electrolyte and quickly rinsed with deionized (DI) water to remove any residue from the surface. The time chart of the growth is shown in Figure 1c. It was confirmed (data is not shown) that the growth without HMTA and heat tend to generate nanoflake-like structure without any one-dimensional (1D) structure. It was shown that HMTA is able to promote the growth of one-dimensional ZnO structure in *c*-axis [26] by cutting off the access of Zn^2+^ ions at the sides of the structure, leaving only the polar (001) face to be exposed to Zn^2+^ ions for further nucleation. As been reported by Kim et al*.*, ZnO nanostructure will not grow on graphene sheets at a growth temperature of 50°C because the activation energy for the nucleation of ZnO nanostructures cannot be achieved at this low temperature [23]. Therefore, higher temperature needs to be applied to achieve the nucleation of ZnO and to increase the hydrolyzation process of HMTA.

The surface morphology, elemental composition, crystallinity, and optical properties of the grown ZnO nanostructures were characterized using scanning electron microscopy (SEM), energy dispersive X-ray spectroscopy (EDX), X-ray diffractometer (XRD), and photoluminescence (PL) spectroscopy with excitation at 325 nm of He-Cd laser, respectively.

## Results and discussion

**Figure 2:**
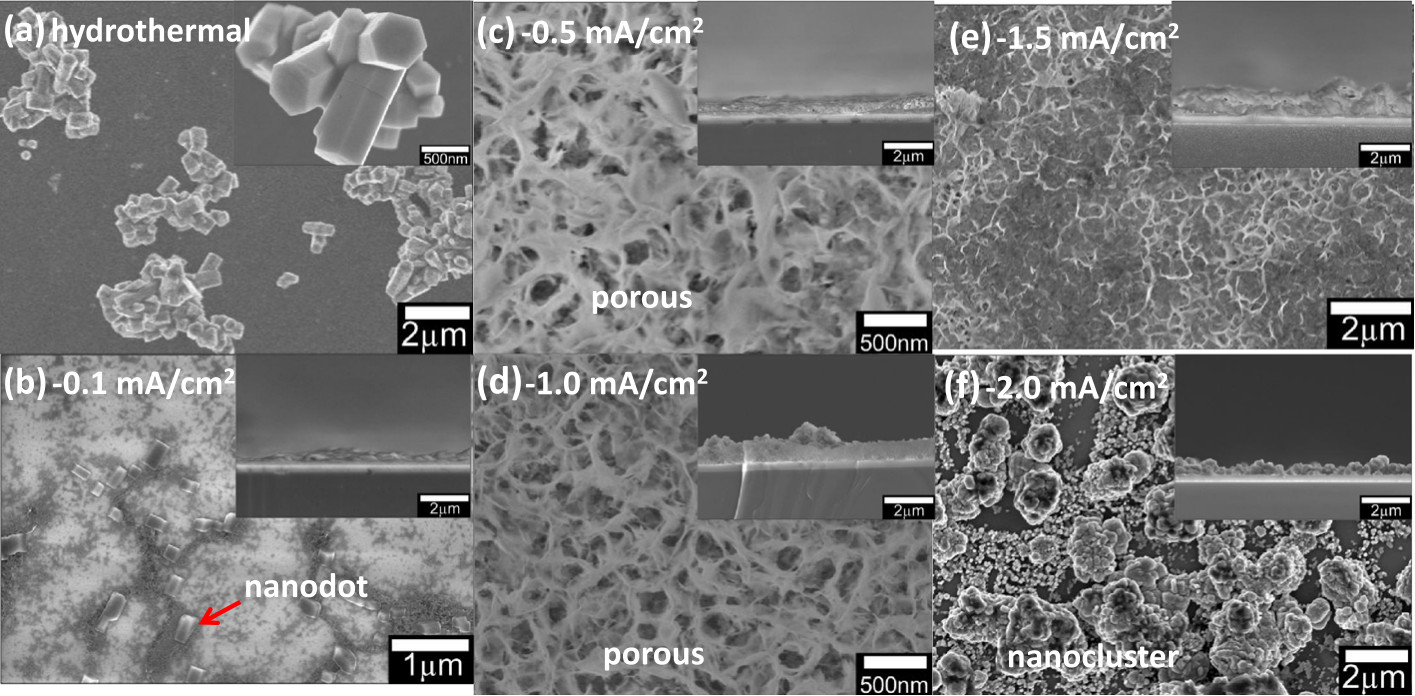
**SEM images of ZnO structures. (a)** Top-view SEM images of ZnO structures grown at a current density of 0.0 mA/cm^2^ (hydrothermal). **(b)**-**(f)** Top-view and cross-sectional SEM images of the initial ZnO structures grown at current densities of -0.1, -0.5, -1.0, -1.5, and -2.0 mA/cm^2^, respectively.

As comparison, firstly, the hydrothermal growth of ZnO using the same composition of electrolyte and temperature was performed in the same setup. As shown in Figure 2a, the grown ZnO nanostructures are nanorod clusters with very low density, and the structures are not vertically aligned. This is not consistent with the results obtained in [23], probably because the growth was not done in a high-pressure container or autoclave. Next, the growth at the preheated stage, i.e., initial growth, was investigated. The growth was performed in a heated mixture of equimolar of Zn (NO_3_)_2_ · 6H_2_O and HMTA with applied current densities of -0.1, -0.5, -1.0, -1.5, and -2.0 mA/cm^2^. As shown in Figure 2b, c, d, e, f, different morphologies of ZnO nucleation structure were observed. The structures seem to be strongly dependent on the applied current density. At low current density of -0.1 mA/cm^2^, a very thin ZnO layer containing nanodot structures was obtained (Figure 2b). When the current densities were increased to −0.5 and −1.0 mA/cm^2^, a ZnO layer with nanoporous-like morphological structures was observed as shown in Figure 2c, d, respectively. The porosity seems to decrease with the increase of current density, where a ZnO layer without porous-like structure was observed at the current density of -1.5 mA/cm^2^ as shown in Figure 2e. At high current density of -2.0 mA/cm^2^, a ZnO layer containing nanocluster structures was observed as shown in Figure 2f. The growth of the vertical nanorods based on those formed seed structures is expected to have been enhanced after the ST point or during the actual growth. Since the reaction of electrolyte is considerably premature at temperatures below 80°C, the crystallinity of the seed structure is not good. This is simply proved by the EDX analysis (data is not shown), where the compositional percentage of zinc (Zn) and oxygen (O) is low which is in the range of 50% to 60% in spite of the additional compositional percentage of O from the SiO_2_ layer.

**Figure 3:**
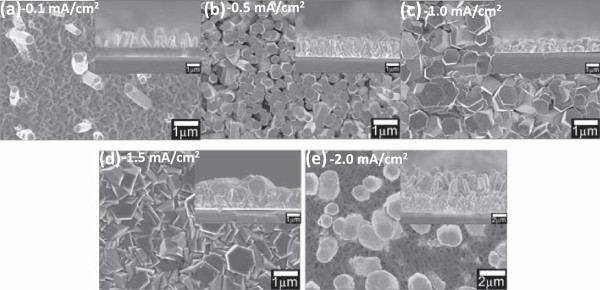
**Top-view and cross-sectional SEM images of final ZnO nanostructures.** The nanostructures were grown at current densities of **(a)** -0.1 mA/cm^2^, **(b)** -0.5 mA/cm^2^, **(c)** -1.0 mA/cm^2^, **(d)** -1.5 mA/cm^2^, **(e)** -2.0 mA/cm^2^.

Finally, the complete growth (i.e., initial plus actual growth) of the ZnO nanostructures according to the time chart shown in Figure 1c in a heated mixture of equimolar of Zn (NO_3_)_2_ · 6H_2_O and HMTA at applied current densities of -0.1, -0.5, -1.0, -1.5, and -2.0 mA/cm^2^ was carried out. Figure 3a, b, c, d, e shows the top-view and cross-sectional SEM images of the grown structures. It is noted that the grown structures show identical morphologies throughout the whole surface area of the graphene. Again, it can be understood that the morphologies of the grown ZnO structures change significantly according to the applied current density. From the EDX analysis, the compositional percentage of Zn and O at current densities of -0.1, -0.5, -1.0, -1.5, and -2.0 mA/cm^2^ was found to be above 90%. At low current density of -0.1 mA/cm^2^, a very small density of nanorods was obtained. These nanorods seem to originate from the ZnO nanodots which were formed during the initial growth. The density of the nanorods was drastically increased at the current density of -0.5 mA/cm^2^ with slight increase in diameter of the nanorod. This is due to the porous-like structures formed during the initial growth which is likely to promote the growth of the nanorods. The same tendency was also reported, where the enhancement of the growth of ZnO nanorods on porous Si was obtained [27]. When the applied current is further increased to -1.0 mA/cm^2^, the diameter of the nanorods increase drastically, generating almost no space between the nanorods. At the current density of -1.5 mA/cm^2^, due to the increase in diameter as well as the increase in chemical reaction, the morphology shows no more well-defined hexagonal structure. At the current density of -2.0 mA/cm^2^, large diameter of rod structure with fairly defined hexagonal shape was observed. These large nanorods seem to originate from the nanoclusters formed during the initial growth. It can be concluded that the shape, diameter, and density of the grown structures are determined by the initial structure formed during the preheated process. Further explanation is presented in the next section, i.e., growth mechanism.

The calculated densities of the nanorods for samples at current densities of -0.1, -0.5, -1.0, -1.5, and -2.0 mA/cm^2^ are estimated to be around 1.84 × 10^7^, 1.37 × 10^9^, 1.24 × 10^8^, 3.42 × 10^7^, and 2.32 × 10^7^ cm^2^, respectively. The density is 1 order larger than the density of the nanorods grown by the hydrothermal process [23] and in the same order with the estimated nanorods grown by the electrochemical process on oxidized graphene layer [25] for the same range of diameter. The current applied in the electrochemical process seems to induce and promote the growth of ZnO nanorods with high density. Table 1 summarizes the density, diameter, length, and average aspect ratio of the grown ZnO and the comparison with other works. High average aspect ratio of more than 2.3 was obtainable with current densities from -0.1 to -0.5 mA/cm^2^.

**Table 1:** Density, diameter, length, and average aspect ratio of the grown ZnO nanorods

	**Current density (mA/cm**^**2**^**)**	**Density (cm**^**2**^**)**	**Diameter of nanorods (nm)**	**Length of nanorods (nm)**	**Average aspect ratio**
This work	-0.1	1.84 × 10^7^	190 to 450	450 to 1,160	2.32
	-0.5	1.37 × 10^9^	260 to 480	840 to 1,160	2.70
	-1.0	1.24 × 10^8^	660 to 1,000	150 to 340	0.28
	-1.5	3.42 × 10^7^	950 to 1,330	200 to 560	0.34
	-2.0	2.32 × 10^7^	570 to 2,030	1,160 to 2,220	1.14
[23]	-	3.00× 10^7^	680	1,400	2.10
[25]	-0.15	5.83× 10^8^	370 to 780	-	-

**Figure 4:**
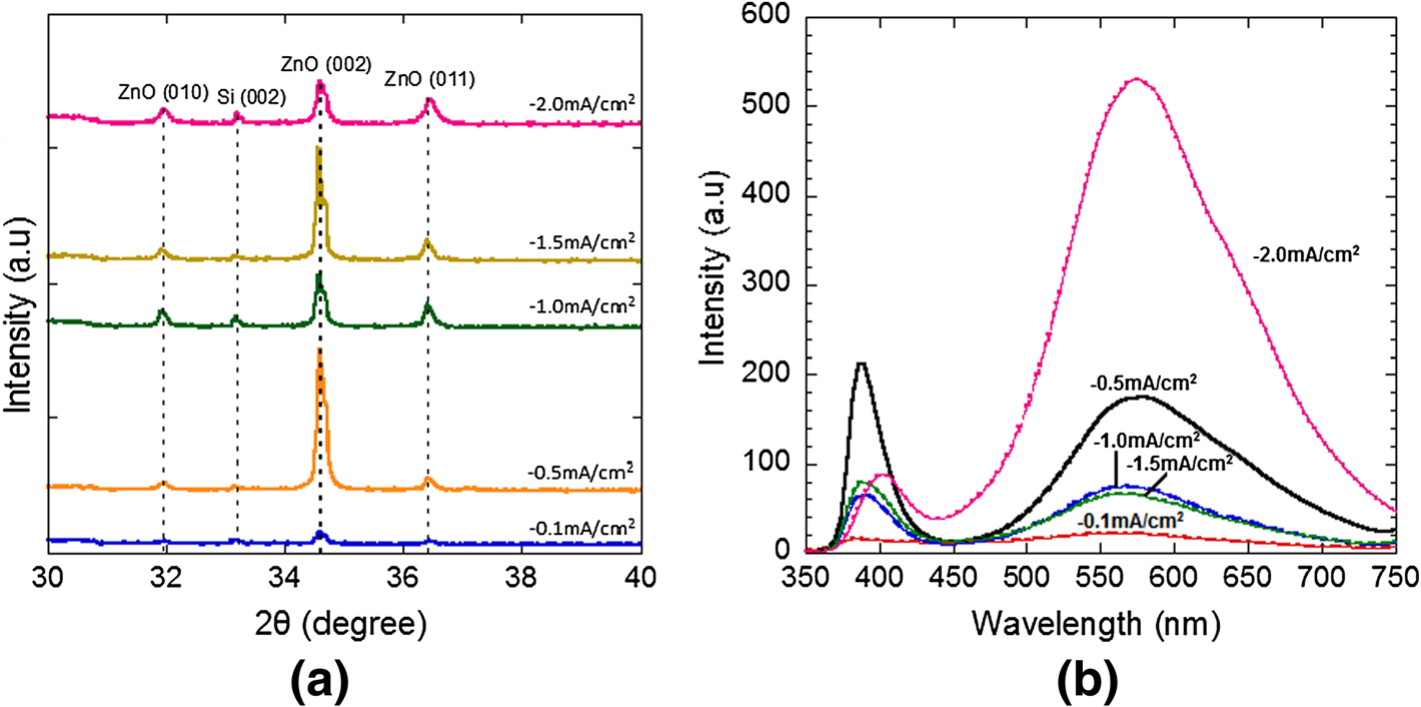
**XRD and RT PL spectra of the grown nanostructures. (a)** XRD spectra and **(b)** RT PL spectra of the grown ZnO nanostructures at different applied current densities.

Figure 4a shows the XRD spectra of the as-grown ZnO nanorods on the SL graphene at different current densities. The diffraction peaks of ZnO at 31.97°, 34.60°, and 36.42° (ICDD 01-075-1526) were recorded which belong to (010), (002), and (011) planes, respectively. These diffraction peaks show that the grown ZnO nanostructures were having hexagonal wurtzite structure. Furthermore, there was also a weak peak at 33.20° which corresponds to the Si (002) diffraction peak (ICDD 01-080-0018). A relatively high peak intensity of the ZnO (002) plane and relatively low peak intensity of ZnO (011) were observed for the samples grown at the current density of -0.5 mA/cm^2^, indicating that the preferred growth orientation of the grown ZnO nanorods is towards the *c*-axis ([001] direction), consistent with the SEM images shown in Figure 3b.

The optical characteristics of the ZnO nanostructures were investigated using RT PL spectroscopy. Figure 4b shows the PL spectra of the ZnO nanostructures deposited on the graphene layers at different current densities. Each RT PL spectrum shows one distinct near-band-edge (NBE) emission peak at 3.210, 3.210, 3.200, 3.200, and 3.080 eV for samples grown at current densities of -0.1, -0.5, -1.0, -1.5, and -2.0 mA/cm^2^, respectively. The full width at half maximum (FWHM) value was estimated to be around 0.20 to 0.37 eV. The strong, sharp NBE emission indicates the high optical quality of the ZnO nanostructures on the graphene layers. It was reported that the PL spectrum at 17 K typically shows five distinct NBE emission peaks with FWHM value of several milli-electron volt [2]. However, only one of these emission peaks which is equal to 3.240 eV was observed in our room-temperature measurement. The other four peaks which tentatively attributed to neutral-donor bound exciton peaks and free exciton peak were not able to be observed. From the PL spectra, no additional exciton peak associated with carbon impurities in carbon-doped ZnO films [28] was observed at 3.356 eV. This suggests that the carbon atoms in the graphene were not incorporated into the ZnO nanorods during their growth. The PL characteristics of the ZnO nanostructures on the graphene layers were almost the same to those of the ZnO nanostructures on single-crystalline substrates such as Si [29,30]. The second band appears in the green region of the visible spectrum at approximately 2.25 to 2.30 eV for the grown samples. The sample at the current density of -2.0 mA/cm^2^ shows the highest green emission than other samples which indicates that there are more defects such as large fraction of O vacancies that have been introduced during the growth process [27,31-33]. The defects are speculated to exist in the seed layer which is formed during the initial growth stage. The observation of the NBE emission peak and weak green emission related to defects suggest high optical quality of the ZnO nanorods grown on the graphene layers. It can be said that the samples grown at −0.5 to −1.5 mA/cm^2^ seem to produce relatively high quality ZnO structures. The control of initial seed layer and further modification of growth procedure may improve the overall structure of ZnO.

### Chemical reaction and growth mechanism

In this work, Zn (NO_3_)_2_ · 6H_2_O is used as source of Zn and O, while HMTA can be considered as a mineralizer to supply extra source of OH^-^ and to define the shape and morphology of the nanorods. The chemical reactions involved are shown by Equations 1 to 7:

(1) $$\mathrm{Zn}{\left({\mathrm{NO}}_{3}\right)}_{2}\to {\mathrm{Zn}}^{2+}+2{{\mathrm{NO}}_{3}}^{-}$$

(2) $${{\mathrm{NO}}_{3}}^{-}+H_{2}O+2e^{-}\to {{\mathrm{NO}}_{2}}^{-}+2{\mathrm{OH}}^{-}$$

(3) $${\mathrm{Zn}}^{2+}+{\mathrm{OH}}^{-}\to \mathrm{Zn}{\left(\mathrm{OH}\right)}_{2}$$

(4) $$\mathrm{Zn}{\left(\mathrm{OH}\right)}^{2-}\to \mathrm{ZnO}+H_{2}O$$

(5) $$C_{6}H_{12}N_{4}+6H_{2}O\to {\mathrm{COH}}_{2}+4{\mathrm{NH}}_{3}$$

(6) $${\mathrm{NH}}_{3}+H_{2}O\to {{\mathrm{NH}}_{4}}^{+}+{\mathrm{OH}}^{-}$$

(7) $$H_{2}O\to ½O_{2}+2H^{+}+2e^{-}.$$

When HMTA was added into Zn (NO_3_)_2_ · 6H_2_O, no precipitation occurred as they are just mixed together initially. With the introduction of temperature, HMTA begins to decompose into ammonia and then Zn(OH)_2_ is produced. The complete decomposition is achieved by continuous heating [34,35]. Finally, it produces ZnO and H_2_O with the presence of OH^−^ and e^−^. HMTA acts as a weak base, slowly hydrolyzing in water and gradually releasing OH^−^ ions [34]. OH^−^ ions are produced during the chemical reaction of HMTA with water as shown in Equations 5 and 6, while e^−^ is obtained from the chemical reaction occurred at the anode as shown in Equation 7. The hydrolyzation of HMTA can be accelerated by increasing the pH of the electrolyte [36].

The vertically aligned nanorods are produced with the help of HMTA. HMTA is a long-chain polymer and a non-polar chelating agent [37]. It will preferably attach to the non-polar facets of the zincite crystal, by cutting off the access of Zn^2+^ ions to the sides of the structure, leaving only the polar [001] face exposed to the Zn^2+^ ions for further nucleation and growth. Hence, HMTA acts as a non-ionic ligand chelate on the non-polar surface of ZnO nanocrystals on the six prismatic side planes of the wurtzite crystal and induces the growth in the *c*-axis [38]. Therefore, HMTA acts more like a shape-inducing polymer surfactant rather than just a buffer [38].

**Figure 5:**
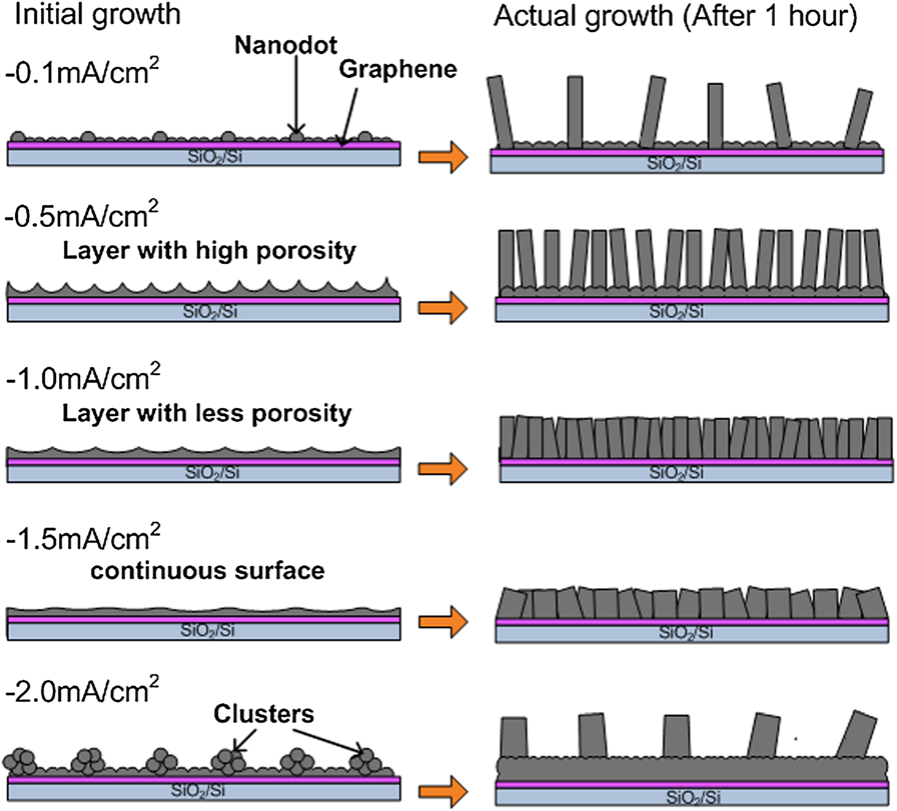
Schematic of the proposed growth mechanism.

The proposed growth mechanism as illustrated in Figure 5 was developed based on Figure 2b, c, d, e, f and Figure 3a, b, c, d, e. The structures formed during the initial growth determine the subsequently grown structures, where a vertical growth was enhanced during the actual growth resulting to the formation of ZnO nanorods. It clearly shows that the applied current density has strongly influenced the morphology of the initial structures. Porous structure helps increase the density of the vertically aligned ZnO nanorods. Cluster structures formed at high current density has resulted to large nanorods. In summary, the growth processes involve two main stages which are the formation of nucleation structure during the initial growth (RT to ST) and the formation of vertical nanorods during the actual growth. As proved by the SEM images, the vertical nanorods do not grow directly on the graphene, but they grow on the nucleation sites formed during the initial growth.

## Conclusions

In conclusion, high density vertically aligned ZnO nanorods has successfully been grown on a single-layer graphene by electrochemical deposition method using heated zinc nitrate hexahydrate and HMTA as the electrolyte. HMTA and heat play a significant role in promoting the formation of hexagonal ZnO nanostructures. The applied current in the electrochemical process plays an important role in inducing the growth of the ZnO nanostructures on the SL graphene as well as in controlling the shape, diameter, and density of the nanostructures. The control of the initial structures and further modification of growth procedure may improve the overall structure of ZnO.

## Competing interests

The authors declare that they have no competing interests.

## Authors’ contributions

NSAA designed and performed the experiments, participated in the characterization and data analysis of FESEM, EDX, XRD, and PL, and prepared the manuscript. MRM participated in the PL characterization. KY participated in the XRD characterization and revision of the manuscript. AMH participated in the monitoring of the experimental work, data analysis, discussion, and revision of the manuscript. All authors read and approved the final manuscript.
